# Diagnostic test accuracies of 4AT items are consistent across the range of baseline cognition: results from two prospective studies

**DOI:** 10.1093/ageing/afag182

**Published:** 2026-06-24

**Authors:** Patrick Hogan, Hannah Cheston, Ophelia Dunne, Dominic Gardner, Amy McWhirter, Mark James Rawle, Imogen Stoodley, Sarah Joanna Richardson, Louise M Allan, Alasdair M J MacLullich, Daniel H J Davis, Alex Tsui

**Affiliations:** Institute of Health Informatics, University College London, UK; Institute of Health Informatics, University College London, UK; Institute of Health Informatics, University College London, UK; Institute of Health Informatics, University College London, UK; Institute of Health Informatics, University College London, UK; Care of the Elderly, Whipps Cross University Hospital, London, England, UK; Institute of Cardiovascular Science, MRC Unit for Lifelong Health and Ageing, 1-19 Torrington Place, London, WC1E 7HB, UK; Institute of Health Informatics, University College London, UK; Translational and Clinical Research Institute, Newcastle University, 3rd floor Biomedical Research Building Campus for Ageing and Vitality, Newcastle upon Tyne, NE4 5PL, UK; Centre for Research in Ageing and Cognitive Health, University of Exeter, St Luke's Campus Heavitree Road, Exeter, EX1 2LU, UK; Geriatric Medicine, University of Edinburgh MED, F1424, Royal Infirmary of Edinburgh 51, Little France Crescent, Edinburgh, EH16 4SA, UK; Institute of Health Informatics, University College London, UK; North Middlesex Hospital, Royal Free London NHS Foundation Trust, London, England, UK; Institute of Health Informatics, University College London, UK; North Middlesex Hospital, Royal Free London NHS Foundation Trust, London, England, UK

**Keywords:** delirium, dementia, diagnosis, older people

## Abstract

**Background:**

Presenting features of delirium can be difficult to distinguish from co-existing dementia phenomenology. While the 4 ‘A’s Test (4AT) is a well validated delirium screening tool across multiple patient populations and healthcare settings, how its diagnostic performance varies across different levels of baseline cognition is unclear.

**Methods:**

This study harmonises data from two prospective cohorts: the *Delirium and Population Health Informatics Cohort* (DELPHIC) and *Delirium and Cognitive Impact in Dementia* (DECIDE). Both cohorts were stratified into tertiles according to baseline cognition. Delirium was defined using DSM-IV (DELPHIC) and DSM-V (DECIDE). 4AT domain scores were operationalised from relevant delirium assessments within each study (MDAS, OSLA, months of the year backwards); item 4 was drawn from assessments of fluctuation. This process yielded an operationalised 4AT. Diagnostic accuracy of the operationalised 4AT was calculated using sensitivity, specificity, and receiver operating characteristics, with pooled estimates for each test domain.

**Results:**

Among 396 unique participants and 2468 assessments across both studies, pooled 4AT sensitivity and specificity were 0.73 and 0.89, respectively. Overall 4AT performance was consistent across cognitive tertiles (sensitivity: high 0.82, middle 0.73, low 0.71; specificity: high 0.91; middle 0.92; low 0.86).

**Conclusions:**

The operationalised 4AT showed good diagnostic performance in all three levels of baseline cognition. Sensitivity would likely have been higher had clinical information to inform 4AT item 4 been available. Overall, however, the findings support the value of bedside 4AT items in detecting delirium in hospitalised patients across the spectrum of baseline cognition.

## Key Points

- The 4AT is diagnostically accurate for delirium, irrespective of baseline cognitive state.
- Some items such as attention and orientation vary in performance depending on cognitive baseline, but the overall accuracy of the 4AT is stable.
- Prospective measures of baseline cognition allow for a more detailed understanding of diagnostic accuracy and support the use of 4AT in unselected hospital populations.

## Introduction

Delirium is a neuropsychiatric syndrome, characterised by acute and fluctuating changes in arousal, orientation and attention, associated with increased length of hospital stay, functional decline, and mortality [1]. Although phenomenologically distinct, delirium and dementia frequently co-present, with delirium superimposed on dementia occurring in nearly half of hospitalised patients with dementia, while 33%–50% of inpatients with delirium have existing dementia [2–4]. Distinguishing delirium from dementia is challenging as bedside assessments are influenced by baseline cognition, while identification of acute change or fluctuations generally require an informant history [5]. Furthermore, ascertainment of acute change and fluctuations may be more intensive in diagnostic accuracy studies than routine clinical practise, affecting apparent test performance.

The 4’A’s Test (4AT) is a well-established screening tool for delirium [6, 7] (www.4at.com). Its strengths lie in its brevity, ability to be administered without specialist training, inclusion of responses for ‘untestable’ patients, and robust performance in routine practise [8, 9]. A meta-analysis of 17 studies found a pooled sensitivity of 0.88 and specificity of 0.88 [7]. Sensitivity is maintained though specificity is marginally lower in people living with dementia; a meta-analysis of five studies reported pooled sensitivity of 0.88 and specificity of 0.79 [6, 10–14].

Previous diagnostic accuracy studies of delirium assessment instruments have typically relied on retrospectively determined dementia status [15]. Such binary classifications provide a limited approximation of test performance variation across the range of baseline cognition. Prospective assessment allows graded quantification of cognitive function, enabling more robust validation of diagnostic accuracy across the cognitive spectrum. Longitudinal data have linked extremes of baseline cognition with increased delirium severity [16]. Clarifying how instruments such as the 4AT perform in unselected populations is therefore critical for enhancing delirium detection.

We used data from two prospective studies, Delirium and Population Health Informatics Cohort (DELPHIC) and Delirium and Cognitive Impact in Dementia (DECIDE), to quantify the association of baseline cognition with 4AT diagnostic performance [17, 18]. Both studies have complementary cognitive measures, allowing mutual validation of 4AT’s overall accuracy, as well as for specific test items.

## Methods

This is a diagnostic accuracy study and meta-analysis using data from the DELPHIC and DECIDE cohorts, detailed protocols for which have been published previously [17, 18]. These are the only population-based longitudinal studies which included prospective assessment of baseline cognition and daily assessment of delirium status during incident hospitalisation. Both studies recruited community-dwelling older people, aged ≥70 in DELPHIC and ≥ 65 in DECIDE.

### Delirium

Participants were assessed at each incident hospitalisation. Delirium domains were quantified using the Memorial Delirium Assessment Scale (MDAS) [19]. Both studies also tested attention using *months of the year backwards* (MOTYB) and arousal using the Observation Scale of Level of Arousal (OSLA) [20]. Delirium status on each day of hospitalisation was defined according to DSM-IV-TR (DELPHIC) or DSM-V (DECIDE) criteria [21, 22].

### 4AT item mapping

The 4AT is scored out of 12 across four domains: *alertness* (0, 4); *AMT4* (0, 1, 2), *attention* (0, 1, 2) and *acute change or fluctuations* (0, 4). Scores ≥4 indicate likely delirium. We did not administer the 4AT directly; rather, we operationalised 4AT items from components of the core assessment, using MOTYB, MDAS and OSLA scores. These domains were mapped to 4AT items, re-scaling continuous variables to give a score out of 4 for each item (Supplementary data, Appendix S1).

Mapping of items was similar between the cohorts with minor methodological differences due to missing data for some items. Two 4AT items have the option of intermediate sub-scores (1 or 2). Sensitivity analysis assigning intermediate scores did not improve test accuracy. Mapping of 4AT items 1–3 was straightforward in that this involved cognitive tests and bedside assessments of level of arousal. Mapping of 4AT item 4 used *fluctuating course* data, but not *acute onset* as this was not separately recorded. This meant information on *acute onset* used to inform the DSM reference standard was not available to score the operationalised 4AT. We estimated the impact of this through sensitivity analyses of diagnostic accuracy using items 1–3 with and without item 4.

### Baseline cognition

Baseline cognitive score (pre-admission) was calculated using a composite of pre-morbid cognitive assessments. DELPHIC participants completed the TICS-m, tests of verbal fluency and selected memory questions from the Addenbrookes Cognitive Examination III. Baseline cognition in DECIDE was ascertained using the Mini-Mental State Examination and verbal fluency tests. The composite cognitive scores in the cohorts were normalised against each other. Each cohort was then divided into tertiles according to baseline cognitive score. This was chosen over regression analysis to aid interpretability and to avoid imposing the assumption that changes in cognitive score map linearly onto diagnostic performance.

### Data analysis

Baseline characteristics were summarised using descriptive statistics and means were compared using the independent samples *t-*test or χ^2^ test where applicable. Operationalised 4AT performance was compared in individuals with high, mid, and low baseline cognition. Diagnostic test accuracy was assessed for the whole 4AT, and for individual domains, by calculating sensitivity, specificity and area under the receiver operating characteristic curve (AUROC), with 95% confidence intervals estimated using bootstrap resampling at the participant level. Meta-analysis of the two studies was performed using the DerSimonian-Laird method to yield pooled sensitivity, specificity and AUROC. Observations for which a full 4AT score could not be calculated due to missing data were excluded. As above, diagnostic test accuracy was tested with and without item 4. We used Python version 3.11 for all analyses except meta-analysis, for which R version 4.1.2 was used.

**Table 1:** Baseline characteristics of study participants admitted to hospital during study periods, by cohort.

	DELPHIC (*n* = 196)	DECIDE (*n* = 200)	*P*-value
Age *mean (SD)*	80.6 (6.4)	82.0 (6.5)	.03
Female sex *N (%)*	106 (54%)	107 (54%)	.91
Years of education *mean (SD)*	11.3 (1.7)	10.4 (2.3)	<.001
Baseline cognition *mean (SD)*	44.6 (11.6)	53 (6.5)	<.001
Delirium cumulative incidence (%)	42.1%	15.7%	<.001
Baseline Barthel *mean (SD)*	18.5 (1.6)	18.1 (3.4)	.42
Frailty *DELPHIC = Frailty index median [IQR]* *DECIDE = Clinical Frailty Scale median [IQR]*	0.22 [0.13, 0.34]	4 [3, 5]	n/a

## Results

We included 396 unique participants, representing 2468 individual assessments (DELPHIC = 1384, DECIDE = 1084). Participants in DECIDE were older than those in DELPHIC (82.0 vs 80.6 years, *P* = .03) with higher baseline cognition (53.0 vs 44.6 standardised score/100, *P* < .001) (Table 1). Delirium incidence within respective study periods were higher in DELPHIC (42.1% vs 15.7%, *P* < .001). Sex distribution was similar in both cohorts (54% women). Participants in DELPHIC had more years in education (11.3 vs 10.4, *P* < .001). Independence with basic activities of daily living, measured by the Barthel index, was similar (18.5 vs 18.1, *P* = .42). Frailty ascertainment differed between the cohorts, but each study included a broad range of frailty states (DELPHIC median FI 0.22, IQR 0.13–0.34; DECIDE median Clinical Frailty Scale 4, IQR 3–5). Characteristics of participants with missing data were not substantially different from those with complete data. Participants excluded from DECIDE due to having no recorded data were frailer and had lower baseline cognitive scores, but these represented just 0.02% of the overall cohort (Supplementary data, Appendix S2).

### Diagnostic utility of 4AT and its domains

**Figure 1:**
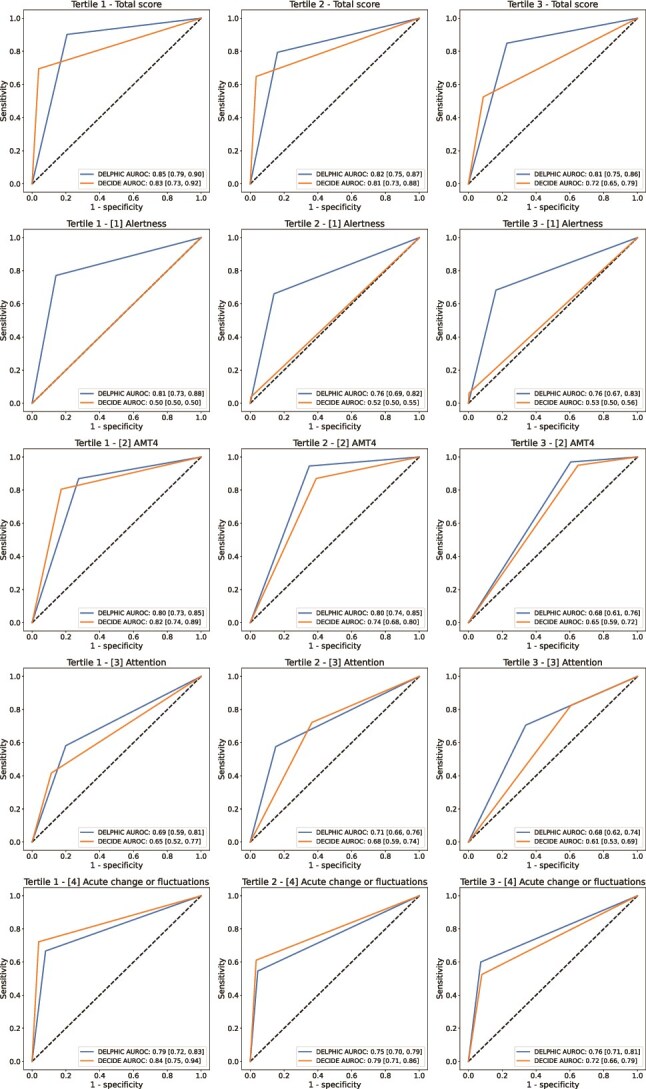
AUROC curves for DECIDE and DELPHIC participants by tertile of baseline cognition and 4AT domain.

**Figure 2:**
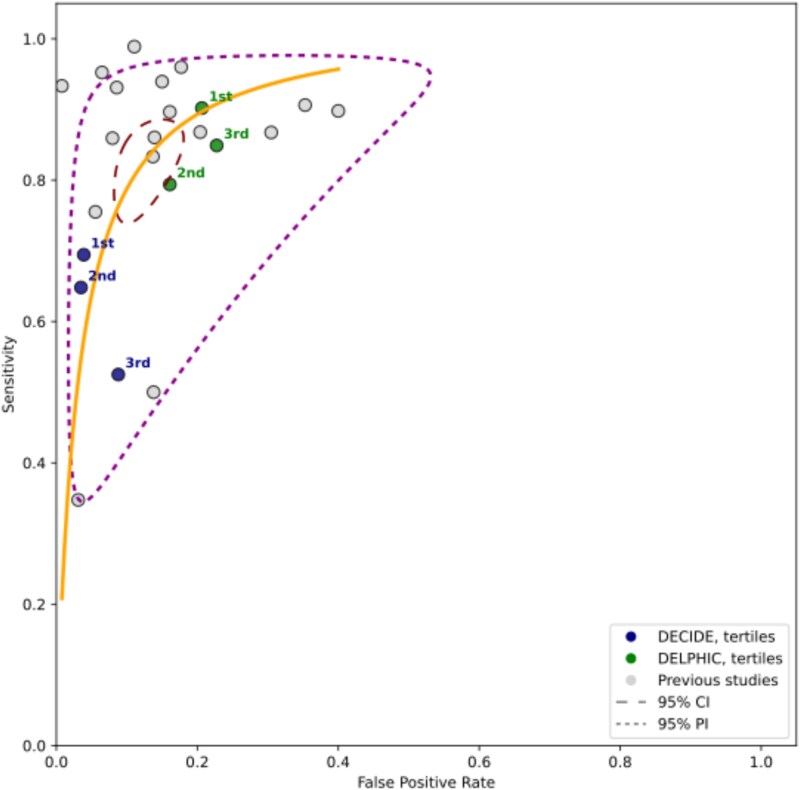
Receiver operating characteristic (ROC) plot comparing prediction and confidence intervals of DELPHIC and DECIDE participants, stratified by baseline cognition tertiles, against previous studies of 4AT detection. Figure adapted with permission from Tieges *et al*. 2021 [7], which includes original references to included studies.

Overall, operationalised 4AT pooled sensitivity and specificity were 0.73 and 0.89 respectively. Performance was broadly consistent across the continuum of baseline cognition; although 4AT had better diagnostic yield in those with higher baseline cognition (AUROC 0.84; Table 2), this declined only slightly with lower cognition (AUROC 0.77; Figure 1). Similarly, sensitivity was higher in those with higher baseline cognition (0.82) compared to those with lowest baseline (0.71). Pooled specificity did not significantly change in any tertile: high 0.91; medium 0.92; low 0.86; Table 2). Set against other studies of hospitalised participants, operationalised 4AT performance was comparable in each cognitive tertile (Figure 2).

**Table 2:** Sensitivity, specificity and area under receiving operating characteristics (AUROC) for DELPHIC and DECIDE participants by 4AT domains, first as whole cohorts, and also stratified by baseline cognition tertiles (1st highest, 2nd mid, 3rd lowest baseline cognition).

		Sensitivity			Specificity			AUROC		
		DELPHIC	DECIDE	Pooled	DELPHIC	DECIDE	Pooled	DELPHIC	DECIDE	Pooled
**Whole cohort**	Total 4AT score	0.85 [0.80, 0.89]	0.60 [0.51, 0.69]	0.74 [0.44, 0.91]	0.80 [0.75, 0.86]	0.95 [0.93, 0.97]	0.90 [0.66, 0.98]	0.83 [0.79, 0.86]	0.77 [0.73, 0.82]	0.80 [0.75, 0.85]
	Alertness	0.70 [0.61, 0.77]	0.04 [0.01, 0.08]	0.24 [0.01, 0.94]	0.85 [0.80, 0.95]	1.00 [0.99, 1.00]	0.98 [0.42, 1.00]	0.78 [0.73, 0.82]	0.52 [0.50, 0.54]	0.65 [0.40, 0.90]
	Orientation (AMT4)	0.94 [0.90, 0.97]	0.89 [0.84, 0.94]	0.92 [0.87, 0.95]	0.60 [0.52, 0.67]	0.63 [0.56, 0.70]	0.62 [0.59, 0.64]	0.77 [0.73, 0.81]	0.76 [0.72, 0.80]	0.77 [0.75, 0.78]
	Attention	0.64 [0.56, 0.71]	0.71 [0.60, 0.80]	0.67 [0.60, 0.74]	0.78 [0.72, 0.83]	0.67 [0.61, 0.73]	0.73 [0.61, 0.82]	0.71 [0.67, 0.75]	0.69 [0.63, 0.74]	0.70 [0.68, 0.72]
	Fluctuation	0.60 [0.54, 0.66]	0.59 [0.51, 0.69]	0.60 [0.59, 0.61]	0.94 [0.91, 0.96]	0.95 [0.94, 0.97]	0.94 [0.93, 0.96]	0.77 [0.74, 0.79]	0.77 [0.73, 0.82]	0.77 [0.75, 0.80]
**1st tertile (highest) baseline cognition**	Total 4AT score	0.90 [0.82, 0.94]	0.69 [0.50, 0.89]	0.82 [0.54, 0.95]	0.79 [0.69, 0.90]	0.96 [0.94, 0.98]	0.91 [0.61, 0.98]	0.85 [0.79, 0.90]	0.83 [0.73, 0.92]	0.84 [0.80, 0.87]
	Alertness	0.77 [0.63, 0.85]	0.00 [0.00, 0.00]	n/a	0.86 [0.75, 0.95]	1.00 [1.00, 1.00]	n/a	0.81 [0.73, 0.88]	0.50 [0.50, 0.50]	n/a
	Orientation (AMT4)	0.87 [0.75, 0.95]	0.81 [0.66, 0.93]	0.84 [0.77, 0.89]	0.72 [0.65, 0.79]	0.83 [0.75, 0.90]	0.78 [0.66, 0.87]	0.80 [0.73, 0.85]	0.82 [0.74, 0.89]	0.81 [0.78, 0.84]
	Attention	0.58 [0.40, 0.79]	0.42 [0.14, 0.67]	0.50 [0.34, 0.66]	0.80 [0.68, 0.92]	0.89 [0.83, 0.93]	0.85 [0.75, 0.91]	0.69 [0.59, 0.81]	0.65 [0.52, 0.77]	0.67 [0.63, 0.72]
	Fluctuation	0.67 [0.51, 0.77]	0.72 [0.53, 0.92]	0.69 [0.64, 0.75]	0.92 [0.86, 0.97]	0.96 [0.94, 0.98]	0.94 [0.89, 0.97]	0.79 [0.72, 0.83]	0.84 [0.75, 0.94]	0.82 [0.77, 0.87]
**2nd tertile (mid) baseline cognition**	Total 4AT score	0.79 [0.70, 0.87]	0.65 [0.50, 0.79]	0.73 [0.56, 0.85]	0.84 [0.75, 0.91]	0.97 [0.94, 0.99]	0.92 [0.70, 0.98]	0.82 [0.75, 0.87]	0.81 [0.73, 0.88]	0.81 [0.78, 0.85]
	Alertness	0.66 [0.53, 0.77]	0.04 [0.00, 0.11]	0.21 [0.01, 0.93]	0.86 [0.78, 0.93]	1.00 [0.99, 1.00]	0.98 [0.48, 1.00]	0.76 [0.69, 0.82]	0.52 [0.50, 0.55]	0.64 [0.40, 0.88]
	Orientation (AMT4)	0.95 [0.89, 0.98]	0.87 [0.78, 0.94]	0.92 [0.81, 0.96]	0.65 [0.53, 0.74]	0.61 [0.48, 0.73]	0.63 [0.59, 0.67]	0.80 [0.74, 0.85]	0.74 [0.68, 0.80]	0.77 [0.71, 0.83]
	Attention	0.58 [0.48, 0.67]	0.72 [0.54, 0.85]	0.65 [0.50, 0.78]	0.85 [0.77, 0.91]	0.63 [0.53, 0.73]	0.76 [0.50, 0.91]	0.71 [0.66, 0.76]	0.68 [0.59, 0.74]	0.70 [0.66, 0.73]
	Fluctuation	0.55 [0.44, 0.63]	0.61 [0.44, 0.76]	0.58 [0.51, 0.64]	0.96 [0.92, 0.98]	0.97 [0.94, 0.99]	0.96 [0.95, 0.97]	0.75 [0.70, 0.79]	0.79 [0.71, 0.86]	0.77 [0.72, 0.82]
**3rd tertile (lowest) baseline cognition**	Total 4AT score	0.85 [0.75, 0.91]	0.52 [0.40, 0.66]	0.71 [0.34, 0.92]	0.77 [0.77, 0.68]	0.91 [0.87, 0.95]	0.86 [0.67, 0.95]	0.81 [0.75, 0.86]	0.72 [0.65, 0.79]	0.77 [0.68, 0.86]
	Alertness	0.68 [0.51, 0.80]	0.06 [0.01, 0.13]	0.27 [0.01, 0.92]	0.84 [0.73, 0.92]	1.00 [0.99, 1.00]	0.97 [0.45, 1.00]	0.76 [0.67, 0.83]	0.52 [0.50, 0.56]	0.65 [0.42, 0.87]
	Orientation (AMT4)	0.97 [0.93, 1.00]	0.95 [0.89, 0.99]	0.96 [0.94, 0.98]	0.39 [0.24, 0.55]	0.35 [0.23, 0.48]	0.37 [0.33, 0.42]	0.68 [0.61, 0.76]	0.65 [0.59, 0.72]	0.67 [0.64, 0.70]
	Attention	0.71 [0.62, 0.80]	0.82 [0.68, 0.96]	0.77 [0.63, 0.87]	0.66 [0.53, 0.76]	0.39 [0.27, 0.52]	0.53 [0.28, 0.77]	0.68 [0.62, 0.74]	0.61 [0.53, 0.69]	0.65 [0.57, 0.72]
	Fluctuation	0.60 [0.50, 0.69]	0.52 [0.40, 0.65]	0.56 [0.49, 0.64]	0.93 [0.88, 0.97]	0.92 [0.89, 0.95]	0.92 [0.92, 0.93]	0.76 [0.71, 0.81]	0.72 [0.66, 0.79]	0.74 [0.70, 0.78]

**Figure 3:**
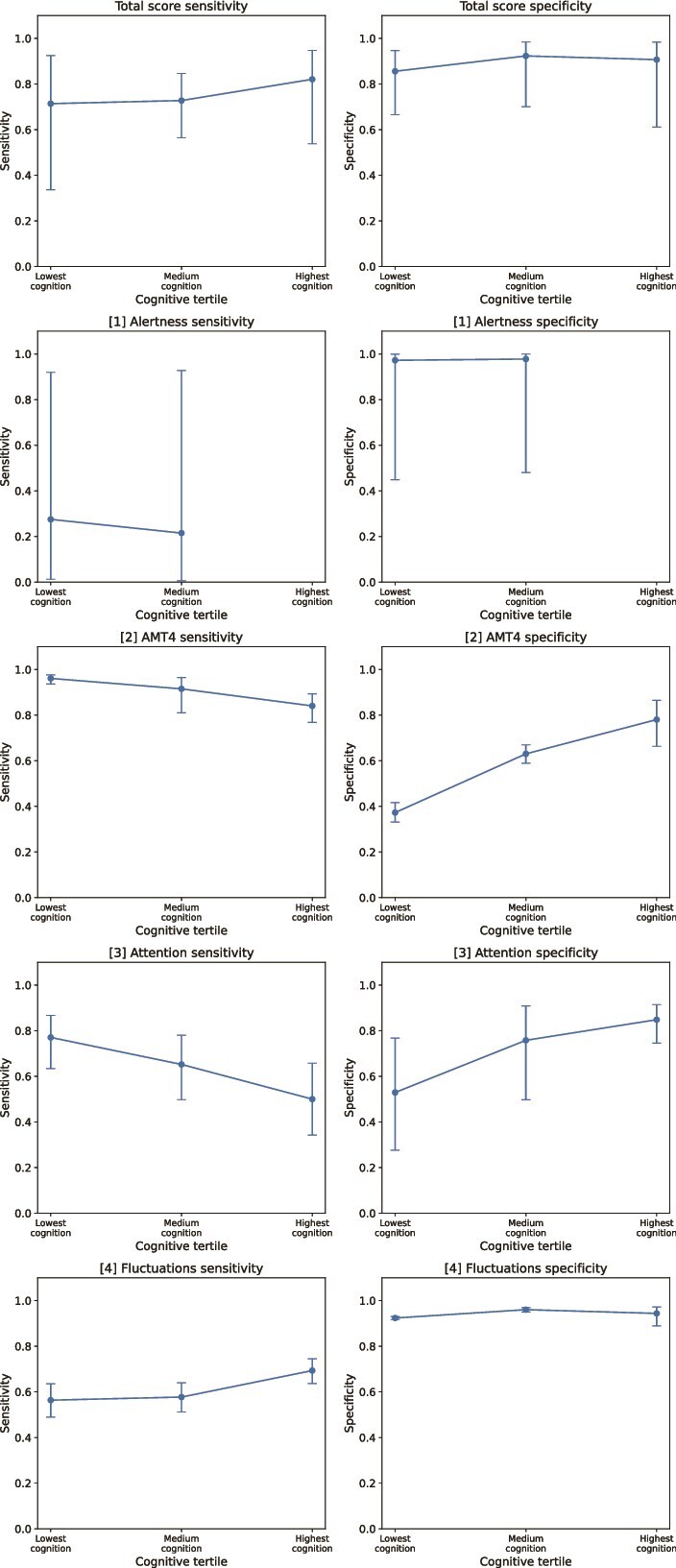
Pooled senitivity and specificity of 4AT and individual components by cognitive tertile.

Diagnostic accuracy of some 4AT items remained largely consistent across groups (Figure 1): *orientation* was highly sensitive across all tertiles, and *fluctuating course* demonstrated similarly high specificity. *Altered alertness* demonstrated high specificity across all tertiles in DELPHIC. However, differences in ascertainment of *alertness* in DECIDE meant calculating pooled specificity was not possible for this item in the highest tertile (Table 2, Figure 3). The greatest variation in item performance occurred for *inattention*. In participants with the lowest baseline cognition, *inattention* demonstrated increased sensitivity but reduced specificity. Conversely, in those with higher baseline cognition, *inattention* was specific, but had lower sensitivity (Table 2, Figure 3). The specificity of *orientation* followed a similar pattern, being lowest in individuals with lower baseline cognition and highest in the top tertile (Figure 1). Sensitivity analysis using *alertness, attention* and *orientation* excluding *fluctuation* led to significant differences in test accuracy in the DECIDE cohort due to lower ascertainment of *altered arousal*. The DELPHIC cohort had lower sensitivity and higher specificity in all tertiles (Supplementary data, Appendix S3).

## Discussion

In two independent prospective cohorts, we found the 4AT to be an effective screening tool for delirium across the cognitive spectrum. Overall diagnostic accuracies did not vary significantly across two independent cohorts, though small item-level differences were demonstrated. Even when stratified by baseline cognition, diagnostic accuracies in these sub-samples were comparable to estimates from whole studies reported in previous meta-analyses [7]. Taken together, the findings support the conclusion that the 4AT appears to have high clinical utility irrespective of prior cognitive baseline.

Study strengths include prospective ascertainment of baseline cognition and objective delirium assessments. Our data are from two large independent samples, supporting generalisability of findings. However, we were unable to capture all participants with delirium if treated in the community. Furthermore, some participants whose first language was not English were not included. Study protocols differed between DELPHIC and DECIDE, with stricter grading of alertness in DECIDE, evidenced by different numbers of participants with abnormal arousal. Nonetheless, overall comparability of the ascertainment framework in both studies was close. The methodology uses a complete case analysis approach which may have inflated test accuracy, though characteristics of participants with missing data was not substantially different from the overall cohorts.

While the cohorts were well matched for age and sex, there was heterogeneity observed in other characteristics, including baseline cognition and delirium incidence which could have affected accuracy of pooled estimates. Additionally, since the 4AT was not directly administered, this was operationalised from other assessment tools, which may have affected accuracy and validity. Similarly, the reference standard delirium assessment differed between the two datasets: DELPHIC used DSM-IV-TR, while DECIDE used DSM-5 criteria; although these frameworks overlap substantially, they are not identical, and this may have introduced further heterogeneity in case ascertainment.

Our findings align well with existing literature. A meta-analysis showed slightly lower 4AT specificity in patients with dementia [14]. Consistent with this, we confirmed that specificity was lower in the bottom tertile, though was still high compared to previous studies. The current study differs from these studies in two key ways. Firstly, dementia is usually ascertained retrospectively from medical notes or informant reports. Dementia diagnosis is also binary, meaning the full spectrum of cognition is not reflected and any such cohort is likely to be unbalanced with fewer patients living with dementia. DELPHIC and DECIDE measured baseline cognition prospectively allowing a more reliable disaggregation of the influence of prior cognitive status.

Although there were differences in individual item performance, the 4AT demonstrated consistent diagnostic accuracies across the cognitive spectrum. The tool maintained high specificity in all groups. While sensitivity was modestly lower in those with reduced baseline cognition, overall performance remained robust. This reinforces the utility of the 4AT as a reliable screening instrument, including in individuals with cognitive impairment. Item-level variations largely reflect expected overlaps between delirium and dementia. For example, *inattention* and *disorientation* were more sensitive but less specific in participants with lower baseline cognition with the converse in those with higher baseline cognition, suggesting they may serve as stronger indicators of delirium when cognition is otherwise intact. It should be noted that item 4—*fluctuation*—did not include information regarding acute onset of symptoms. This represents a limitation which may have led to lower sensitivity as seen on sensitivity analysis excluding *fluctuation*. Nevertheless, *fluctuation* performed well across all tertiles, supporting the inclusion of non-higher order cognitive features in delirium screening tools, particularly for patients with dementia. While no single feature is diagnostic, and some loss of specificity is inevitable in those with dementia, the 4AT overall showed strong performance. This is consistent with studies of 4AT performance in other conditions such as stroke, Parkinson’s disease, traumatic brain injury, and hearing/visual impairment; in which the 4AT has been demonstrated to have good diagnostic accuracy [23–26]. Results are also similar to studies of the 3D-CAM which have shown slightly lower sensitivity and specificity in those with cognitive impairment, though specificity may be maintained in the intensive care setting using the CAM-ICU [27, 28].

In conclusion, the 4AT is a valid screening tool for delirium in individuals of all cognitive baselines. Cognitive features perform well in those with higher baseline cognition, while non-cognitive features are more useful for those with lower baseline cognition. Our findings emphasise that while baseline cognition may influence the performance of individual 4AT items, it does not substantially affect overall diagnostic accuracy. The 4AT is a robust and practical tool for delirium detection across the full spectrum of cognitive function in hospitalised patients.

## Supplementary Material

Supplementary_materials_afag182

## Data Availability

Complete deidentified participant data, along with study protocols, consent forms, and case report forms are available through the Dementias Platform UK Data Portal: https://portal.dementiasplatform.uk/.
