# Supplementary material for: Diagnostic test accuracies of 4AT items are consistent across the range of baseline cognition: results from two prospective studies
Source: Age Ageing. 2026 Jun 24;55(6):afag182. doi: 10.1093/ageing/afag182 (PMC13293242; doi:10.1093/ageing/afag182)
Supplement: Supplementary_materials_afag182 [file supplementary_materials_afag182.docx]

# **Diagnostic test accuracies of 4AT items are consistent across the range of baseline cognition: results from two prospective studies**

**Appendix 1**: Harmonised 4AT domain score from DELPHIC and DECIDE primary data

| **4AT Domain** | **4AT score** | **DELPHIC** | **DECIDE** |
| --- | --- | --- | --- |
| **1. Alertness** | 0 | MDAS 9 < 2  OSLA eye opening < 2  OSLA eye contact < 2  OSLA posture ≤ 2  OSLA movement < 2 | MDAS 9 < 3  OSLA eye opening < 3  OSLA eye contact < 2  OSLA posture ≤ 2  OSLA movement < 3 |
|  | 4 | MDAS 9 ≥ 2  OSLA eye opening ≥ 2  OSLA eye contact ≥ 2  OSLA posture = 3  OSLA movement ≥ 2 | MDAS 9 = 3  OSLA eye opening ≥ 3  OSLA eye contact ≥ 2  OSLA posture = 3  OSLA movement ≥ 3 |
| **2. Orientation/ AMT4** | 0 | MDAS 2 = 0 | MDAS 2 = 0 |
|  | 1 | MDAS 2 = 1 or 2 | MDAS 2 = 1 or 2 |
|  | 2 | MDAS 2 = 3 | MDAS 2 = 3 |
| **3. Attention** | 0 | MOTYB ≥ 7  MDAS 4 = 0  MDAS 5 = 0 | MOTYB ≥ 7  MDAS 4 = 0  MDAS 5 = 0 |
|  | 1 | MOTYB > 0 and < 7  MDAS 4 = 1  MDAS 5 = 1 | MOTYB < 7  MDAS 4 = 1  MDAS 5 = 1 |
|  | 2 | MOTYB = 0  MDAS 4 ≥ 2  MDAS 5 ≥ 2 | MOTYB = untestable  MDAS 4 ≥ 2  MDAS 5 ≥ 2 |
| **4. Fluctuation** | 0 | Absence of motor fluctuations  Absence of arousal fluctuations  No change in daily OSLA or MDAS  MDAS 10 ≤ 1 | Absence of fluctuations  MDAS 10 = 0 |
|  | 4 | Presence of motor fluctuations  Presence of arousal fluctuations  Change in daily OSLA or MDAS  MDAS 10 ≥ 2 | Presence of fluctuations  MDAS 10 > 0 |

**Appendix 2**: characteristics of participants with missing data

|  | **DELPHIC missing data**  **(n = 110)** | **DELPHIC no data (n = 13)** | **DECIDE missing data**  **(n = 105)** | **DECIDE no data**  **(n = 4)** |
| --- | --- | --- | --- | --- |
| Age *mean (SD)* | 81.9 (6.4) | 81.2 (5.7) | 83.5 (6.8) | 84.0 (8.8) |
| Female sex *N (%)* | 62 (56%) | 7 (54%) | 53 (50%) | 2 (50%) |
| Years of education *mean (SD)* | 11.1 (1.9) | 11.6 (1.5) | 10.2 (2.1) | 9.8 (2.1) |
| Baseline cognition *mean (SD)* | 43.3 (11.5) | 47.5 (8.7) | 50 (7.5) | 37.6 (12.6) |
| Baseline Barthel *mean (SD)* | 17.6 (2.9) | 18.2 (1.4) | 17.6 (3.5) | 11.3 (4.3) |
| Frailty  *DELPHIC = Frailty index median [IQR]*  *DECIDE = Clinical Frailty Scale median [IQR]* | 0.25 [0.16, 0.38] | 0.22 [0.19, 0.31] | 5 [4, 6] | 7 [6.8, 7] |

**Appendix 3:** sensitivity analysis for d4AT items 1-3 only

|  | **Sensitivity** | | |  | **Specificity** | | |  | **AUROC** | | |
| --- | --- | --- | --- | --- | --- | --- | --- | --- | --- | --- | --- |
|  | DELPHIC | DECIDE | Pooled |  | DELPHIC | DECIDE | Pooled |  | DELPHIC | DECIDE | Pooled |
| **Whole cohort** | 0.76 | 0.05 | 0.74 |  | 0.84 | 1.0 | 0.90 |  | 0.80 | 0.52 | 0.80 |
| **1^st^ tertile (highest)** | 0.83 | 1.0 | n/a |  | 0.85 | 0.0 | n/a |  | 0.84 | 0.50 | n/a |
| **2^nd^ tertile (mid)** | 0.69 | 0.04 | 0.23 |  | 0.85 | 1.0 | 0.98 |  | 0.85 | 0.52 | 0.64 |
| **3^rd^ tertile (lowest)** | 0.76 | 0.09 | 0.35 |  | 0.82 | 0.99 | 0.96 |  | 0.79 | 0.54 | 0.66 |
